# Dual-Time Point [^68^Ga]Ga-PSMA-11 PET/CT Hybrid Imaging for Staging and Restaging of Prostate Cancer

**DOI:** 10.3390/cancers12102788

**Published:** 2020-09-28

**Authors:** Manuela A. Hoffmann, Hans-Georg Buchholz, Helmut J Wieler, Florian Rosar, Matthias Miederer, Nicolas Fischer, Mathias Schreckenberger

**Affiliations:** 1 Department of Occupational Health & Safety, Federal Ministry of Defense, 53123 Bonn, Germany; 2 Clinic of Nuclear Medicine, Johannes Gutenberg-University, 55101 Mainz, Germany; hans-georg.buchholz@unimedizin-mainz.de (H.-G.B.); florian.rosar@uks.eu (F.R.); matthias.miederer@unimedizin-mainz.de (M.M.); mathias.schreckenberger@unimedizin-mainz.de (M.S.); 3 Clinic of Nuclear Medicine, Bundeswehr Central Hospital, 56072 Koblenz, Germany; helmut.wieler@web.de; 4 Department of Nuclear Medicine, Saarland University Medical Center, 66421 Homburg, Germany; 5 Department of Urology, University of Cologne, 50937 Cologne, Germany; nicolas.fischer@uk-koeln.de

**Keywords:** [^68^Ga]Ga-PSMA PET/CT, prostate cancer, dual-time point imaging, delayed imaging, biphasic imaging, lesion positivity rate

## Abstract

**Simple Summary:**

Early diagnosis and tumor characterization of prostate cancer (PCa) are important for accurate treatment. [^68^Ga]Ga-PSMA-11 PET/CT turns out to constitute a major step toward improved diagnostic procedures to detect primary, recurrent, and metastatic PCa. The aim of our study is to evaluate the effect of a second imaging modality for the staging and restaging of PCa by possibly detecting additional PCa lesions due to the well-known increase of PSMA uptake over time. There was a significant increase in tracer uptake on delayed images in comparison to early [^68^Ga]Ga-PSMA-11 PET/CT in our study, but the lesion positivity rate was comparable. However, in a few individual cases, additional delayed scans provided an information advantage in PCa lesion detection. The findings of our study are likely to be of major interest to clinicians as well as to researchers defining the algorithms that are necessary to implement this promising method with its specific tracer into clinical routine.

**Abstract:**

Routine [^68^Ga]Ga-PSMA-11 PET/CT (one hour post-injection) has been shown to accurately detect prostate cancer (PCa) lesions. The goal of this study is to evaluate the benefit of a dual-time point imaging modality for the staging and restaging of PCa patients. Biphasic [^68^Ga]Ga-PSMA-11 PET/CT of 233 patients, who underwent early and late scans (one/three hours post-injection), were retrospectively studied. Tumor uptake and biphasic lesion detection for 215 biochemically recurrent patients previously treated for localized PCa (prostatectomized patients (P-P)/irradiated patients (P-I) and 18 patients suspected of having primary PCa (P-T) were separately evaluated. Late [^68^Ga]Ga-PSMA-11 PET/CT imaging detected 554 PCa lesions in 114 P-P patients, 187 PCa lesions in 33 P-I patients, and 47 PCa lesions in 13 P-T patients. Most patients (106+32 P-P/P-I, 13 P-T) showed no additional PCa lesions. However, 11 PSMA-avid lesions were only detected in delayed images, and 33 lesions were confirmed as malignant by a SUVmax increase. The mean SUVmax of pelvic lymph node metastases was 25% higher (*p* < 0.001) comparing early and late PET/CT. High positivity rates from routine [^68^Ga]Ga-PSMA-11 PET/CT for the staging and restaging of PCa patients were demonstrated. There was no decisive influence of additional late imaging with PCa lesion detection on therapeutic decisions. However, in a few individual cases, additional delayed scans provided an information advantage in PCa lesion detection due to higher tracer uptake and improved contrast.

## 1. Introduction

Prostate cancer (PCa) is the most commonly diagnosed cancer with an incidence of 1.276 million worldwide in 2018 [1]. Early diagnosis, accurate staging, and tumor characterization are critical for selection of optimal therapy. Molecular imaging with positron-emission tomography (PET) is regarded as a relevant diagnostic approach and has found its way into the guidelines of the European Association of Urology (EAU guidelines) on PCa [2,3]. The prostate-specific membrane antigen (PSMA) is a transmembrane glycoprotein that is significantly overexpressed in most prostate adenocarcinomas, compared with other PSMA-expressing tissues [4]. After many years of preclinical research on PSMA ligands, a breakthrough was achieved in 2011 with the clinical introduction of Glu-NH-CO-NH-Lys(Ahx)-{^68^Ga-(N,N′-bis-[2-hydroxy-5-(carboxyethyl)benzyl]ethylen-ediamine-N,N′-diacetic-acid)}([^68^Ga]Ga-HBED-CC-PSMA or [^68^Ga]Ga-PSMA-11) as a ^68^Gallium (^68^Ga)-labeled PSMA-targeted radioligand for PET/computed tomography (CT) [5,6]. PSMA PET/CT offers an appealing combination of PCa specificity and high sensitivity at low tumor volumes [7]. Sensitive and specific imaging is a fundamental requirement for the definition of the target volume in radiotherapy planning. One of the main limitations of both CT and magnetic resonance imaging (MRI) for lymph node (LN) staging is their limited capability to detect metastatic clusters in normal sized nodes; and microscopic LNM are often not enlarged [8,9]. The accurate assessment of locoregional LN metastases (LNM) is much more sensitive with PSMA PET/CT than with MRI [9]. Whereas PSMA PET/CT can detect an LNM of diameter of 3 mm, MRI can generally only identify pathological LN when they show aberrant anatomical characteristics such as a short-axis diameter >1 cm and/or non-oval shape. However, up to 80% of metastasis-involved nodes are smaller than this threshold limit that is typically used in clinical practice [10]. Meta-analytical data for the traditional CT and MRI imaging approaches suggest sensitivity of only 39–42% and specificity of 82% [10]. Since normal lymphatic or retroperitoneal fatty tissue does not demonstrate PSMA expression, metastatic LNs can be detected with a favorable lesion-to-background ratio. [^68^Ga]Ga-PSMA PET/CT imaging has been shown to accurately detect PCa lesions for LNM [11,12]. These characteristics have led to the evolution of PSMA PET/CT as an important diagnostic tool in nuclear medicine [7,9,13]. In 130 patients with intermediate to high-risk PCa, a sensitivity of 65.9% and a specificity of 98.9% for LN staging using [^68^Ga]Ga-PSMA-11 PET/CT was reported by Maurer et al. [12]. 

It has been described that PCa metastases demonstrate an increase of PSMA ligand uptake over time [5,14]. According to the Heidelberg group [5], 70% of PCa lesions have increased uptake and contrast three hours (h) post-injectionem (p.i.) compared to one h p.i. Clarification of the special situation of pelvic LNM and the possible impact of additional delayed imaging for salvage or primary therapy would be important for improved clinical decision making. 

The goal of our study is to evaluate the effect of a second (late) imaging modality for the restaging and initial staging of patients with recurrent PCa, using additional findings in the abdominopelvic area based on the well-known increase of PSMA uptake over time.

## 2. Results

### 2.1. Overall Lesion Positivity Rate

A positivity rate in 147 out of 215 restaging patients (68%) (mean prostate-specific antigen (PSA) serum level 19.2 ± 82.5 ng/mL) and in 13 out of 18 primary staging patients (72%) (mean PSA 39.1 ± 67.5 ng/mL) was shown by [^68^Ga]Ga-PSMA-11 PET/CT. At least one lesion suspect for malignancy was detected in these patients. This retrospective study includes 147 restaging patients (prostatectomized patients (P-P) and irradiated patients (P-I)) and 13 staging patients (patients suspected of having primary PCa (P-T)), both with PSMA-positive findings (Table 1). To ensure accurate statistical analysis and a homogenous patient population, the biochemically recurrent (BC)-patients, previously treated by radical prostatectomy (patient group P-P) and those previously treated by irradiation (patient group P-I) were separately evaluated according to the definition protocol of BC patients [15]. 

#### 2.1.1. Lesion Positivity Rate Post-Prostatectomized (P-P)

- Baseline: 551 lesions in 114 patients

In this subgroup (P-P and baseline PET/CT), the detection efficacy was 27% (33) for PSA levels of 0.2 to <0.5 ng/mL and 32% (25), 70% (27), 77% (43), and 90% (50) for PSA levels of 0.5 to <1 ng/mL, 1 to <2 ng/mL, 2 to <5 ng/mL, and ≥5 ng/mL, respectively (*p* < 0.001) (Table 2).

Patients with a PSMA-positive scan showed local recurrence in 24% (27/114) and metastases in 90%. Of the patients with metastases, 39% exhibited local metastases and 30% exhibited distant metastases, and 31% showed both. In 70% of the patients, LNM were detected, 78% of which were pelvic LNM (Table 2).

- Delayed: 554 lesions in 114 patients

Late imaging (3 h after intravenous injection (p.i.)) showed no difference in the detection efficacy when considering the patients without separate division of the number of lesions (Table 2). 

#### 2.1.2. Lesion Positivity Rate Post-Irradiated (P-I)

- Baseline: 186 lesions in 33 patients

This subgroup (P-I and baseline PET/CT) showed a detection efficacy rate of 100% for PSA levels of 2 to <5 ng/mL and 94% for PSA levels of ≥5 ng/mL, respectively. Local recurrence was detected in 79% and metastases were detected in 67%. A total of 42% of the patients showed LNM, while 80% of them showed pelvic LNM. Due to the small number of patients, a statistical analysis would not have given meaningful results.

- Delayed: 187 lesions in 33 patients

The detection efficacy rates 3 h p.i. showed the same results as baseline images. 

#### 2.1.3. Lesion Positivity Rate Pre-Therapy (P-T)

All patients (13) with PSMA-positive lesions showed histopathologically (biopsy-proven) adenocarcinoma PCa.

- Baseline: 47 lesions in 13 patients

In this subgroup (P-T and baseline PET/CT), the detection efficacy was shown in 69% for PSA levels of >4 to <50 ng/mL and in 100% for PSA levels of ≥50 ng/mL. Primary tumor lesions in the prostate were detected in 100%, metastases were detected in 38%, and LNM were detected in 31%, of which pelvic LNM were shown in 75%. Statistical analysis was not done due to the small patient number.

- Delayed: 47 lesions in 13 patients

No difference in the detection efficacy was shown in late compared to baseline imaging. 

### 2.2. Impact of Delayed Imaging on Lesion Positivity Rate

**Figure 1:**
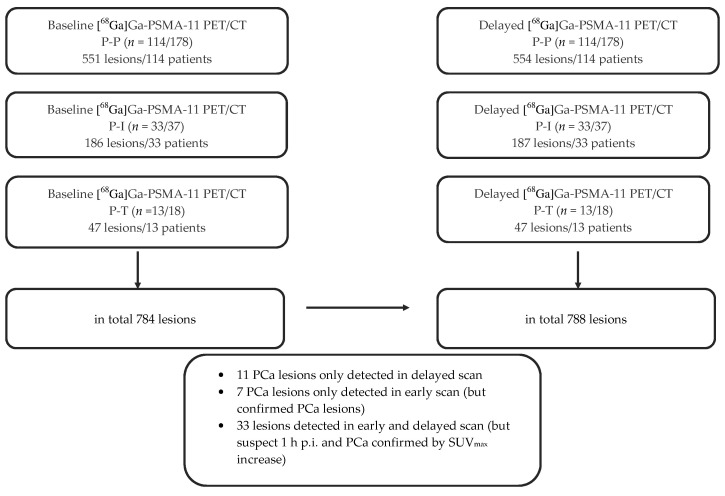
Flow chart showing baseline and delayed [^68^Ga]Ga-PSMA-11 PET/CT results regarding LPR in patients.

**Figure 2:**
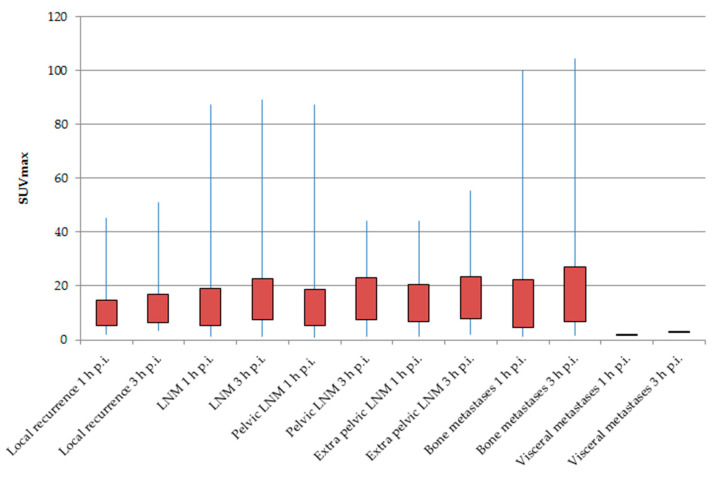
Comparison of baseline and delayed [^68^Ga]Ga-PSMA-11 positron-emission tomography/computed tomography (PET/CT) regarding tracer uptake of prostate-specific membrane antigen (PSMA)-positive lesions (P-P).

A combination of results from both scans (baseline and delayed [^68^Ga]Ga-PSMA-11 PET/CT) revealed a total of 788 lesions (554 P-P, 187 P-I, 47 P-T) (Figure 1 and Figure 2).

#### 2.2.1. Impact of Delayed Imaging on Lesion Positivity Rate P-P

A total of 551 lesions in 114 patients were detected on early scans. Twenty-nine of these lesions were local recurrent findings, and 326 were LNM (262 pelvic LNM and 64 extra-pelvic LNM). In delayed images, 328 LNM (262 pelvic LNM and 66 extra-pelvic LNM) were found. A total of 106 patients showed no additional malignant lesions in late images. Three lesions were only found in the late imaging (two extra-pelvic LNM and one bone metastasis). Comparison of tracer accumulation in pathologic lesions between baseline and delayed scans was statistically significant (*p* < 0.001 pelvic LNM, bone metastases), but this increase in maximum standardized uptake value (SUVmax) did not correspond to a significant influence of late images on the lesion positivity rates (LPR) (Table 3, Figure 1).

#### 2.2.2. Impact of Delayed Imaging on Lesion Positivity Rate P-I

In 33 patients, 186 lesions were found in baseline PET/CT (Figure 1). By comparison, the 33 patients showed 187 findings in late imaging. No additional PCa lesions were shown in 32 patients. In total, a single lesion in one patient was noted 3 h p.i. (one local recurrent PCa lesion in the prostate bed). The comparison of SUVmax in pathologic lesions between early and late images was statistically significant (*p* = 0.008 pelvic LNM). However, there was no significant impact of delayed imaging on LPR.

#### 2.2.3. Impact of Delayed Imaging on Lesion Positivity Rate P-T

All 13 [^68^Ga]Ga-PSMA-11 PET/CT-positive patients showed 47 lesions (Figure 1). No additional PCa lesions were identified by late imaging.

#### 2.2.4. Total Comparison of Biphasic Lesion Detection

Eleven patients with discordant results showed 51 discordant lesions. All unclear lesions (33 lesions moderately suspicious of malignancy) detected by [^68^Ga]Ga-PSMA-11 PET/CT on standard imaging (1 h p.i.) could be clarified by additional late images (3 h p.i.). The decision to classify the lesions as malignant was made on the basis of various criteria such as a higher tracer uptake with increased SUVmax in the late images compared to the early images (Table 3), and an improved contrast as well as presentation of the lesions with a more focal character. The assessment was carried out by nuclear medicine and radiological specialists with several years of diagnostic experience with regard to the analysis of oncologic PET/CT imaging of PCa foci.

- LPR on PET, but not on CT:

By comparison of PET and CT imaging separately, nine LNM with high PSMA avidity were detected on PET, but these were not suspect of malignancy on CT alone.

- Lesions only detected on early imaging:

Seven PSMA-avid lesions (five LNM and two bone metastases) were only shown on early imaging (two P-P and zero P-I). These findings could not be confirmed as PCa lesions on delayed images.

- Lesions only detected on delayed imaging:

In this study, 11 lesions suspicious of malignancy were detected exclusively by delayed imaging (eight P-P and one P-I).

- Additional impact of delayed imaging:

A total of 33 PSMA-avid lesions (of which 15 were LNM, eight were bone metastases, and five were lesions in the prostate bed) suspected of being malignant were confirmed as malignant by increased tracer uptake in the delayed scans.

- No additional impact of delayed imaging/concordant lesions:

In 222 of 233 evaluable patients (95%), the baseline PET/CT and the delayed PET/CT were concordant.

- Time dependency of LPR:

PSMA avidity in pelvic LNM was related more often to scan time than in other metastases (e.g., extra-pelvic LNM, bone metastases, visceral metastases) (*p* < 0.001). Comparing early and late PET/CT imaging, the mean SUVmax of pelvic LNM was 25% higher (*p* < 0.001) and the mean SUVmax of extra-pelvic LNM was 14% higher (*p* = 0.003), respectively.

### 2.3. SUVmax

An increase of tracer accumulation over time was observed in patient groups P-P, P-I, and P-T. The SUV_max_ values of the detected sites of PCa lesions of the late scans were higher than those of the baseline scans. Overall, the SUV_max_ values of tumor lesions in the late PET/CT scans was higher in 22.1% (P-P), 22.5% (P-I), and 17.8% (P-T) than the SUV_max_ values in the baseline scans (each *p* < 0.001) (Table 3). 

#### 2.3.1. SUV_max_ of Malignant Lesions (P-P)

The Wilcoxon test showed a statistically significant difference in SUVmax between baseline and delayed scans. SUVmax was the highest in bone metastases (mean + standard deviation/SD: 17.0 ± 20.2 3 h p.i.; *p* < 0.001) and lowest in local recurrence in the prostate bed (10.1 ± 9.5 1 h p.i./11.7 ± 10.7 3 h p.i.; *p* < 0.001). The SUVmax of LNM showed high values of 15.2 ± 15.5 3 h p.i. for pelvic LNM (*p* < 0.001) and 15.6 ± 15.6 3 h p.i. for extra-pelvic LNM (*p* < 0.005) (Table 3, Figure 2). 

#### 2.3.2. SUV_max_ of Malignant Lesions (P-I)

The greatest increase of tracer uptake over time was seen in bone metastases: at 1 h p.i. SUVmax 24.3 ± 44.6 vs. at 3 h p.i. SUVmax 29.3 ± 48.6 (*p* = 0.002). 

#### 2.3.3. SUV_max_ of Malignant Lesions (P-T)

In P-T patients, an increase of tracer accumulation was also observed in bone metastases, but these data were not statistically significant (*p* = 0.068).

### 2.4. Gleason Score

The LPR showed a clear differentiation depending on the primary histological starting situation, which is expressed by the evaluation system for determining the aggressiveness of PCa. According to previous studies, PCa with a Gleason score (GS) of 7b (4 + 3) has a significantly worse prognosis than PCa with a GS of 7a (3 + 4). For this reason, 7a is classified as grade group 2 and 7b is classified as grade group 3, although they belong to the same group of intermediate-risk PCa. In our study, 11% of the PSMA-positive subgroup P-P was previously categorized as low-risk PCa (GS < 7) with grade 1 according to the International Society of Urological Pathology (ISUP) and intermediate-risk with grade 2 (GS 7a), whereas a categorization of PCa grade 3 to grade 5 (GS 7b, 8 and >8; intermediate up to high-risk) was found in 89% (Table 4) [3,16,17]. By comparison of the LNM-LPR of grade group 1 to 2 PCa-patients (GS ≤ 7a) with that of grade group 3 to 5 (GS ≥7b), a statistically significant difference (*p* = 0.029) was noted (12% vs. 88% respectively) (Table 4). 

When comparing grade 1 to 2 PCa patients (GS ≤ 7a) with grade 3 to 5 PCa patients (GS ≥ 7b) in the subgroup P-I, we evaluated an LNM-LPR in 10% for grade 1 to 2 and in 80% for grade 3 to 5. In the subgroup P-T, all PSMA-avid LNM belong to grade group 3 to 5 (GS ≥ 7b). However, these results were not statistically significant.

### 2.5. Subpopulation

Based on EAU guidelines, which suggest the examination of PSMA PET/CT in patients with PSA serum values of ≥1 ng/mL and based on the definition of BC (PSA is >0.2 ng/mL in prostatectomized patients), we highlighted and examined the patient collective of restaging patients (P-P) in the range from 0.2 to <1 ng/mL as particularly assessable [3,15]. In baseline PET/CT, 33% (58/178) of patients (P-P) showed PSA values in the range of 0.2 to <1 ng/mL, of which 29% were PSMA-positive and 14% showed local metastases (*p* < 0.001) (Table 5). There was no difference in the results determined by delayed PET/CT images. 

In one of our previous studies, we determined an optimal PSA cutoff level of 1.24 ng/mL for distinguishing between positive and negative PSMA PET/CT results for BC patients after primary prostatectomy (P-P) [13]. In this study, P-P patients in baseline shots, with PSA < 1.24 ng/mL, showed an overall positivity in 34% (24/71), PSMA-avid local metastases in 13% (9/71), and distant metastases in 10% (7/71), compared to 84% (90/107), 29% (31/107), and 22% (24/107) in patients with PSA ≥ 1.24 ng/mL (*p* < 0.001) (Table 5). The results were comparable in delayed images.

## 3. Discussion

In this study, the biphasic [^68^Ga]Ga-PSMA-11 PET/CT of 233 patients was retrospectively studied. A total of 178 prostatectomized patients and 37 irradiated patients as well as 18 pre-therapy patients were assessed, and their data (e.g. tumor uptake, biphasic LPR) were separately evaluated. As reported in other studies, we also found high LPR from baseline [^68^Ga]Ga-PSMA-11 PET/CT for the staging (72%) and restaging (68%) of PCa patients [11,13,18]. 

A recently published prospective, randomized, multi-center study from Australia [9] including 300 men with biopsy-proven PCa found that [^68^Ga]Ga-PSMA PET/CT yielded 92% accuracy in identifying those with distant metastatic or pelvic nodal disease compared with 65% accuracy from traditional imaging (CT, bone scan). Furthermore, conventional imaging had more equivocal findings, fewer management changes, and higher radiation doses (19.2 mSv vs. 8.4 mSv; *p* < 0.001) [9]. In addition to improving detection, PSMA PET/CT will have a significant impact on a patient’s treatment plan and disease management in future guidelines [9,11].

Several acquisition protocols with different acquisition times, including early dynamic to 3 h p.i. imaging, have been proposed for [^68^Ga]Ga-PSMA PET/CT studies [18,19,20,21,22]. Time activity curves acquired from PCa lesions showed a continuously increasing tracer accumulation during early dynamic PET acquisition, which also supports the essential role of additional late imaging [22]. The addition of delayed scans has been considered to offer substantial advantages for the discrimination of PCa versus non-PCa lesions, as the malignant foci usually show a further increase in tracer accumulation on the late scans. Benign lesions, on the other hand, usually show a decrease in SUV [18,19,20,21,22]. The optimal time point for the various currently available tracers for PSMA PET/CT imaging and the potential of additional late images have been and are currently being investigated. In the present study, we evaluated the incremental value of [^68^Ga]Ga-PSMA-11 PET delayed imaging, especially abdominopelvic imaging.

As described by previous studies, there is a significant increase in SUVmax of PCa lesions on delayed images when evaluating dual time point [^68^Ga]Ga-PSMA-11 PET/CT imaging [18,21,23,24]. Beheshti et al. reported that this increase relates to suspicious lesions (*p* < 0.001) in the prostate bed (11.6 ± 8.2 to 14.8 ± 1.0) as well as to LNs (9.7 ± 5.9 to 12.3 ± 8.8) [23]. Nevertheless, lesions’ tracer accumulation on early imaging has been sufficient for diagnosis [23], which is consistent with our results.

Afshar-Oromieh et al. reported a mixed pattern of tracer behavior (increase/decrease of SUVmax in metastases) in the same patient in 11.6% (8/69 patients), whereas six of 69 patients (8.7%) showed a consistent decrease in metastatic uptake [20]. We did not find similar results in our patient group. Another study showed a SUVmax decrease in 26 out of 157 lesions [23]. Beheshti et al. reported [23] an increase of SUVmax over time in most lesions, which was also in agreement with our findings. Delayed images could confirm malignancy of 33 moderately PSMA-avid lesions, which were suspicious of being malignant on early scans, due to the increase of tracer uptake. However, some of the findings were ambiguous (11 lesions only detected in delayed scan, seven lesions only detected in early scan), but all of them were characteristic for PCa in follow-up (such as PSMA PET/CT, PSMA PET/MRI, CT, MRI), which support the results of a previous study that demonstrated [^68^Ga]Ga-PSMA PET/CT to be a helpful tool to determine malignancy in ambiguous lesions [24]. These different results might be explained by various tumor cell biologies. Afshar et al. suspect that individual lesions may have a decreased rate of internalization of the PSMA ligand [20]. We speculate that miscellaneous and mixed patient populations may also account for at least some of the different findings that have been reported in the literature. In the large patient group with recurrent PCa, the role of primary treatment (e.g., prostatectomy, radiation therapy) could be an important factor as well.

In our study, the clinically most important group (P-P) of recurrent PCa-patients (*n* = 114/178) showed no significant difference in LPR (551 lesions early vs. 554 late). However, a statistically significant increase of tumor uptake in PCa lesions detected by baseline PET/CT compared with delayed PET/CT was shown. As described by previous PSMA PET studies, the standardized uptake values (SUVs) of LNs are significantly higher 3 h p.i. than 1 h p.i., and nearly all LNM of PCa show high PSMA expression [18,25]. The increase of SUVmax values between baseline and delayed scan in our study also did not significantly raise the number of pathological findings in the P-I and in the P-T-groups, especially for LN lesions, which are of important clinical interest for therapy planning. Due to the fact that microscopic LNs uploaded with metastatic tumor cells are frequently non-enlarged, LN staging and restaging by CT and MRI alone is limited [8], and PSMA PET/CT or PET/MRI is preferred [9,11,12,26]. In a series of PCa patients, the authors found 72% [25] of [^68^Ga]Ga-PSMA-avid LNs to be metastatic in normal-sized LNs (<1 cm) [25,26]. Our data demonstrated nine LNM with high PSMA avidity on PET, which showed no signs of malignancy on CT alone. Aside from the overexpressed PSMA avidity in prostate tumor cells, the LPR of microscopic LNs could improve in the near future as the next generation of scanners (including time-of-flight technique) results in increased spatial resolution, which—compared to the older scanner systems—leads to a higher contrast as well as a higher intrinsic sensitivity [24,27]. 

A just published comprehensive literature search [28], including nine retrospective and two prospective studies, reported detection rates of [^68^Ga]Ga-PSMA-PET in recurrent patients for PSA <0.2 ng/mL, for 0.2–0.49 ng/mL, and for PSA 0.5 to <1.0 ng/mL ranged from 11% to 50%, 20% to 73%, and 25% to 88%. Our results match those of Luiting et al. We had LPR values of 27% for 0.2 to <0.5 ng/mL and 32% for 0.5 to <1.0 ng/mL. The subgroup of patients with PSA < 0.2 ng/mL was excluded in our study, because they do not belong to BC patients per definition [15]. The authors [28] observed high specificity rates of [^68^Ga]Ga-PSMA-PET imaging for pelvic LNM detection in primary staging as well as in restaging, while sensitivity was modest, and they concluded that [^68^Ga]Ga-PSMA PET has a high impact in patient management concerning the salvage setting [11,28,29]. In our study, we found LNM in 70% of the PSMA-avid metastases, 78% of which were pelvic LNM in restaging patients (P-P). In primary tumor staging, LNM were detected in 31%, of which pelvic LNM were shown in 75%. Previous studies report the dynamic uptake of PSMA in ganglia (e.g., celiac ganglia) [18,30]. However, none of the patients in the present study showed PSMA-positive celiac ganglia, neither 1 h p.i. nor 3 h p.i.

The impact of delayed imaging in our patient groups (P-P, P-I, P-T) was limited due to the lack of significantly increased rates of pathological findings 3 h p.i. Our findings were consistent with the results of a study by Derlin et al. [31] using [^68^Ga]Ga-THP-PSMA, who also found that delayed imaging did not increase the number of detected metastases significantly (two out of 99 patients). In contrast, Afshar-Oromieh et al. [32], using [^68^Ga]Ga-PSMA-11, found a 3-h delay as an optimal time point for imaging, as the majority of cancer lesions could be detected then. However, their patient cohort was very small (*n* = 4). It is known from early pharmacokinetic studies that the background activity decreases significantly between 1 and 3 h p.i., resulting in an improved tumor/background (T/B) ratio [5]. However, in the present study, these higher T/B ratios or contrasts to lesions’ tracer accumulation did not result in significantly higher LPR on delayed images, compared to other authors [21]. It must be taken into account here that due to the ^68^Ga’s relatively short half-life of 68 minutes, the count statistics in the 3-hour measurement are significantly lower. Most PC lesions (97%) in our study were already detected early in the imaging process, which sheds some doubt on the need for a second late examination in clinical routine. However, sometimes, it can also be useful to perform late images, e.g., if the effect of urinary activity in assessing pelvic PCa lesions remains unclear in baseline scans [26,33]. In this setting, imaging with ^18^F-labeled compounds (PSMA-based radiopharmaceuticals such as [^18^F]PSMA-1007) should be considered [34], since it offers advantages in late imaging due to the longer half-life of 110 minutes and the significantly lower positron range with improved resolution and the detection of small LNs. However, with regard to a theranostic approach, therapies with [^177^Lu]Lu-PSMA make a pre-therapeutic PET/CT with [^68^Ga]Ga-PSMA appear more meaningful [6,35]. From our theranostic point of view, dual-time point imaging definitely has an important teaching value. The results of our study do not really support a routine performance of supplementary late images for every PSMA PET/CT examination. However, delayed imaging is useful to confirm or rule out a suspicious abnormality seen in early images in individual cases. An important point to emphasize is that additional late images are useful for clearing up unclear lesions whose signs of malignancy would lead to a change in the therapeutic approach.

## 4. Materials and Methods

### 4.1. Patient Characteristics

The clinical characteristics of the 233 included patients are summarized in Table 1. From 2015 to 2018, 233 patients for staging and restaging were retrospectively evaluated. [^68^Ga]Ga-PSMA-11 PET/CT of 215 BC patients who had previously undergone either radical prostatectomy (178, patient group P-P, PSA elevation to >0.2 ng/mL by definition) or radiation therapy (37, patient group P-I, PSA elevation to >2 ng/mL above nadir by definition) and 18 patients with elevated PSA serum level of >4.0 ng/mL, highly suspicious of having primary PCa (group P-T) were separately assessed [15]. All PSMA-positive lesions of P-T were histopathologically confirmed as PCa by biopsy. In case of BC (P-P, P-I), when the biopsy or surgery of PSMA-avid lesions was not possible or considered too invasive for the patients (e.g., bone metastases), we rated the increase of PSA before therapy and decrease after therapy as a tumor confirmation and marker. Additionally, we have included the findings of follow-up examinations (such as PSMA PET/CT, PSMA PET/MRI, CT, MRI).

This retrospective study was done in accordance with the Declaration of Helsinki. All reported investigations were conducted according to the national regulations (German Medical Products Act, AMG § 13.2b). The protocol was approved by the Ethics Committee of Laek Rlp (2018-13390). All patients signed an informed consent (including participation in the study and for evaluation and publication of their anonymized data).

### 4.2. Imaging Protocol and Analysis

^68^Ga-labeled PSMA ligand, Glu-urea-Lys(Ahx)-HBED-CC ([^68^Ga]Ga-PSMA-11), was synthesized using sterile methods as previously described by Eder et al. [36]. The included patients underwent imaging on a Biograph 64 TruePoint (True V HD) PET/CT scanner (Siemens/Erlangen/Germany) 60 ± 10 min (whole body; baseline scan) p.i. of 195.5 ± 48.3 MBq (median activity: 193 MBq, range: 97–299 MBq) and 180 ± 10 min. p.i. (pelvic, abdominal, and suspicious regions; delayed scan). The following parameters were used: three-dimensional acquisition mode (168 × 168); acquisition time of three min. per bed position; axial field of view (FOV): 21.8 cm; random, scatter, and decay correction; ordered-subsets expectation maximization method (OSEM) for PET image reconstruction (two iterations, 14 subsets, Gaussian filtering, 4.2 mm transaxial resolution, full-width at half-maximum). Attenuation corrections were performed using the low-dose non-enhanced CT data (120 kV, 20–60 mAs, CT transverse scan-field 50 cm, 70 cm extended FOV, resolution 1.0 s, 0.6 mm) or the contrast-enhanced CT data (140 kV, 100–400 mAs, dose modulation). The images were assessed by nuclear medicine clinicians and radiologists (each with more than 5 years experience in PET/CT imaging) and reviewed visually in consensus by two board-certified nuclear medicine clinicians and one board-certified radiologist. The term "lesion positivity rate" is used based on the imaging result and its interpretation by the nuclear medicine and radiological expert team in relation to the PSMA-positive tumor lesions. Any lesion with an increased radiotracer uptake (measured with SUV_max_) above physiological uptake was considered suspicious of malignancy, and biphasic lesion detection (baseline and delayed images) was taken into account. If no consensus could be found between the board-certified nuclear medicine clinicians and the board-certified radiologist, these lesions were classified as moderately suspicious of malignancy. However, in this case, all the experts classified the lesions as abnormal and probably malignant. SUV_max_ of PSMA-avid lesions detected by baseline and by delayed scan were compared. There have been extensive efforts to develop quantitative criteria for the analysis of oncologic PET images. The measures proposed are based on the SUV in a certain volume of interest (VOI) enclosing the lesion. The principle of the SUV was introduced by Strauss and Conti [37]. For a defined VOI, the mean SUV value (SUV_mean_) of all included pixels is usually calculated as a representative measure of tracer uptake. As a result of the VOI definition dependence, the SUV_mean_ suffers from a limited reproducibility. To overcome this problem, the SUV_max_ has been introduced, which is the maximal SUV value in the lesion. Thus, we have only used the SUV_max_ in the present study. LNM were divided into two groups based on their location (pelvic LNM: iliac and/or pararectal) and extra-pelvic distant LNM (retroperitoneal and/or above the iliac bifurcation).

### 4.3. Statistical Analysis

Data were analyzed using IPM SPSS Statistics version 23.0 (IBM Corporation, Ehningen, Germany). First, variables were tested for normal distribution using the Shapiro–Wilk test. To compare normally distributed values of two patient groups, Student’s-*t*-test was used. The Mann–Whitney U-test was used for non-normally distributed continuous variables. For further analysis, we evaluated PSA-stratified LPR and evaluated categorical differences by Chi-square test and Pearson correlation. For comparing values of baseline and delayed imaging, i.e., SUV_max_ of PET-positive lesions, the Wilcoxon-signed-rank-test was used. Mean and SD are given if normality was observed. Additionally, for non-normal distributed variables, median and range were evaluated. *p* values < 0.05 were considered statistically significant.

## 5. Conclusions

Although there was a significant increase in SUVmax on delayed images in pelvic and extra-pelvic LNM in comparison to early [^68^Ga]Ga-PSMA PET/CT in our study, the LPR was comparable, especially in the assessment of small subcentimeter pelvic PCa lesions in patients with multiple metastases.

The few additional findings, respectively, the confirmed lesions in the late images, had no effect on staging or restaging of PCa, as they did not lead to any modification of the final interpretation or TNM classification and did not change patient management. However, in a few individual cases, additional late scans provided an information advantage in PCa lesion detection due to a higher tracer uptake and an improved contrast.

## Figures and Tables

**Table 1:** Patient characteristics.

Characteristics (*n*)	Parameters
Number of patients	233
Age (y) (233)	
Median	72
Range	47–85
Mean ± SD	70.3 ± 7.3
Primary Gleason score (228)	
≤6 (low risk + grade group 1)	16
7a, 7b (intermediate risk + grade group 2 + 3)	96
8 (high risk + grade group 4)	33
>8 (high risk + grade group 5)	83
PSA (ng/mL) (233)	
Median	2.32
Range	0.2–960
Mean ± SD	15.1 ± 71.9
Prior treatment of primary tumor (233)	
Surgery (radical prostatectomy)	178
Radiotherapy and other	37
Primary staging (pre-therapy)	18
Further treatment	
Anti-androgen therapy (x/233)	101
Lesion positivity rate (160/233)	68.7%
Restaging (PET/CT-positive/total)	147/215
Primary staging (PET/CT-positive/total)	13/18

Abbreviations: PSA, prostate-specific antigen; SD, standard deviation; *n*, number of patients; y, year.

**Table 2:** PET/CT findings: Lesion positivity rate (LPR) post-prostatectomized (P-P) related to different PSA values.

PSA (ng/mL)	0.2–<0.5	0.5–<1.0	1.0–<2.0	2.0–<5.0	≥5.0	Chi^2^, *p*
Number (x/178) post-prostatectomized patients	33	25	27	43	50	
PET/CT-positive (x/114)	9	8	19	33	45	*r* = 0.507; *p* < 0.001
Lesion positivity rate	27.3%	32.0%	70.4%	76.6%	90.0%	
Regions:						
Local recurrence	2	0	5	6	14	*r* = 0.236; *p* = 0.01
Metastases	7	8	16	31	41	*r* = 0.471; *p* < 0.001
Site of metastases:						*r* = 0.459; *p* < 0.001
Local metastases	4	4	9	11	12	
Distant metastases	0	4	4	10	13	
Local + distant metastases	3	0	3	10	16	
Number of metastases:						*r* = 0.536; *p* < 0.001
Single metastases	3	6	7	7	2	
Multiple metastases	4	2	9	24	39	
Lymph node metastases (LNM)	7	4	12	21	28	*r* = 0.296; *p* = 0.001
Site of LNM:						*r* = 0.297; *p* < 0.042
Pelvic LNM	6	4	10	17	19	
Extra-pelvic LNM	0	0	1	1	2	
Pelvic + extra-pelvic LNM	1	0	1	3	7	
Bone metastases	2	4	5	18	24	*r* = 0.355; *p* < 0.001
Visceral metastases	0	0	1	1	4	*r* = 0.153; *p* = 0.352 *

* Fisher’s exact test. Abbreviations: PSA, prostate-specific antigen; LNM, lymph node metastases; *p* < 0.05 is considered significant; *r*, Pearson correlation coefficient.

**Table 3:** Comparison of baseline and delayed PET/CT P-P related to tracer uptake of PSMA-positive lesions.

Tumor Location	Number of Patients (x/178)	PET/CT-Positive Patients (x/114)	Number of PSMA-Positive Lesions	SUV_max_ Mean ± SD Range	Wilcoxon *p*
Local recurrence 1 h p.i.	27	27	29	10.1 ± 9.5/2.1–45.2	
Local recurrence 3 h p.i.	27	27	29	11.7 ± 10.7/3.6–50.9	*p* < 0.001
LNM 1 h p.i.	72	72	326	12.2 ± 13.6/1.1–87.2	
LNM 3 h p.i.	72	72	328	15.0 ± 15.1/1.2–89.2	*p* < 0.001
Pelvic LNM 1 h p.i.	68	68	262	12.0 ± 13.7/0.9–87.2	
Pelvic LNM 3 h p.i.	68	68	262	15.2 ± 15.5/1.2–89.2	*p* < 0.001
Extra-pelvic LNM 1 h p.i.	16	16	64	13.8 ± 13.8/1.2–44.2	
Extra-pelvic LNM 3 h p.i.	16	16	66	15.6 ± 15.6/2.0–55.5	*p* < 0.005
Bone metastases 1 h p.i.	53	53	195	13.5 ± 18.0/1.3–100.1	
Bone metastases 3 h p.i.	53	53	196	17.0 ± 20.2/1.8–104.5	*p* < 0.001
Visceral metastases 1 h p.i.	1	1	1	2.1	
Visceral metastases 3 h p.i.	1	1	1	3.2	

Abbreviations: LNM, lymph node metastases; SUVmax, maximum standardized uptake value; *p* < 0.05 is considered significant.

**Table 4:** Gleason score in relation to [^68^Ga]Ga-PSMA-11 PET/CT LPR P-P.

*n* = 178	GS < 7 (11)	GS 7a (33)	GS 7b (51)	GS 8 (26)	GS > 8 (57)	Chi^2^ *r*, *p* Value
PSMA-positive (xx/114)	1	11	38	25	39	0.326; *p* < 0.001
Local recurrence (xx/27)	1	4	5	6	11	0.112; *p* = 0.446
Metastases (xx/103)	0	9	34	23	37	0.346; *p* < 0.001
LNM (xx/68)	0	8	22	17	21	0.186; *p* = 0.029

Abbreviations: GS, Gleason score; *n*, number of patients; *p* < 0.05 is considered significant; *r*, Pearson correlation coefficient.

**Table 5:** Baseline PET/CT: LPR P-P of different subgroups related to PSA subgroups.

**PSA Range (ng/mL)**	**Overall Positivity**	**Chi^2^*p*/*r* Value**		**Single Metastases**		**Multiple Metastases**		**Chi^2^*p*/*r* Value**
0.2 to <1 (58)	17 (29.3%)			9 (15.5%)		6 (10.3%)		
<1.24 (71)	24 (33.8%)			12 (16.9%)		9 (12.7%)		
≥1.24 (107)	90 (84.1%)			13 (12.1%)		69 (64.5%)		
Total (178)	114 (64%)	*p* < 0.001 *r* 0.513		25		78		*p* < 0.001 *r* 0.522
**PSA Range (ng/mL)**	**Local Recurrence**	***p*/*r* Value**	**Local Metastases**		**Distant Metastases**		**Local + Distant Metastases**	***p*/*r* Value**
0.2 to <1 (58)	2 (3.4%)		8 (13.8%)		4 (6.9%)		3 (5.2%)	
<1.24 (71)	3 (4.2%)		9 (12.7%)		7 (9.9%)		5 (7.0%)	
≥1.24 (107)	24 (22.4%)		31 (29.0%)		24 (22.4%)		27 (25.2%)	
Total (178)	27	*p* = 0.001 *r* 0.249	40		31		32	*p* < 0.001 *r* 0.412

Abbreviations: PSA, prostate-specific-antigen; *p* < 0.05 is considered significant; *r*, Pearson correlation coefficient.
